# C-Reactive Protein and TGF-α Predict Psychological Distress at Two Years of Follow-Up in Healthy Adolescent Boys: The Fit Futures Study

**DOI:** 10.3389/fpsyg.2022.823420

**Published:** 2022-03-11

**Authors:** Jonas Linkas, Luai Awad Ahmed, Gabor Csifcsak, Nina Emaus, Anne-Sofie Furberg, Guri Grimnes, Gunn Pettersen, Kamilla Rognmo, Tore Christoffersen

**Affiliations:** ^1^Department of Health and Care Sciences, UiT The Arctic University of Norway, Narvik, Norway; ^2^Institute of Public Health, College of Medicine and Health Sciences, United Arab Emirates University, Al Ain, United Arab Emirates; ^3^Department of Psychology, UiT The Arctic University of Norway, Tromsø, Norway; ^4^Department of Health and Care Sciences, UiT The Arctic University of Norway, Tromsø, Norway; ^5^Faculty of Health and Care Sciences, Molde University College, Molde, Norway; ^6^Division of Internal Medicine, University Hospital of North Norway, Tromsø, Norway; ^7^Institute of Clinical Medicine, UiT The Arctic University of Norway, Tromsø, Norway; ^8^School of Sport Sciences, UiT The Arctic University of Norway, Alta, Norway; ^9^Department of Research and Development, Finnmark Hospital Trust, Alta, Norway

**Keywords:** psychological distress, inflammatory markers, depressive symptoms, anxiety symptoms, adolescence

## Abstract

**Objective:**

The scarcity of research on associations between inflammatory markers and symptoms of depression and anxiety during adolescence has yielded inconsistent results. Further, not all studies have controlled for potential confounders. We explored the associations between baseline inflammatory markers and psychological distress including moderators at follow-up in a Norwegian adolescent population sample.

**Methods:**

Data was derived from 373 girls and 294 boys aged 15–18 years at baseline, in the Fit Futures Study, a large-scale 2-year follow-up study on adolescent health. Baseline data was gathered from 2010 to 2011 and follow-up data from 2012 to 2013. Psychological distress was measured with Hopkins Symptom Checklist (HSCL-10). Serum levels of the following inflammatory markers were measured: C-reactive protein (CRP), Interleukin 6 (IL-6), Transforming growth factor alpha (TGF-α), Tumor necrosis factor alpha variant 1 (TRANCE), and variant 2 (TWEAK). Independent associations between baseline inflammatory markers and HSCL-10 at follow-up were explored by linear regressions, in sex-stratified analyses.

**Results:**

In girls, analyses showed positive associations between all inflammatory markers and HSCL-10, except for TRANCE. However, all associations were non-significant in crude as well as in adjusted analyses. In boys, CRP (*p* = 0.03) and TGF-α (*p* < 0.01) showed significant associations with HSCL-10, that remained significant after adjustment. Additionally, moderators were found. In boys, CRP was associated with HSCL-10 in those with high body fat and those being physical inactive, and the association between TWEAK and HSCL-10 was dependent upon sleep duration.

**Conclusion:**

There were significant prospective associations between CRP, TFG-α, and HSCL-10 in boys aged 15–18 years at baseline.

## Introduction

Adolescents who experience psychological distress have increased risk of developing mental disorders later in life (Silva et al., 2020). “Psychological distress,” defined as “state of emotional suffering characterized by symptoms of depression and anxiety” (Mirowsky and Ross, 2002), is commonly reported in younger age groups. In Norway, self-reported psychological distress during high school years was recently reported to be 31 and 12% among girls and boys, respectively (Bakken, 2019). To improve prevention of psychological distress, better knowledge about biological risk factors acting during adolescence is needed. One potential risk factor is low-grade chronic inflammation, which has been linked to mental illness in adulthood.

Interestingly, one in three adults with major depressive disorder (MDD) have elevated levels of inflammatory markers (Leighton et al., 2018), and the average levels of inflammatory markers in MDD patients are higher than in controls (Raison et al., 2006; Howren et al., 2009; Dowlati et al., 2010; Liu et al., 2012; Osimo et al., 2020). Patients with inflammatory conditions have higher risk for MDD compared to controls (Leighton et al., 2018), and up to 50% of patients receiving therapeutic administration of the cytokine interferon-α develop MDD (Raison et al., 2006). There is also preliminary evidence suggesting that patients with general anxiety disorders show increased levels of inflammatory markers compared to controls (Vogelzangs et al., 2013; Michopoulos et al., 2017; Costello et al., 2019). Inflammatory cytokines access the brain through several routes and interact with pathophysiologic domains relevant to depression, including alterations in the metabolism of dopamine, serotonin, and norepinephrine in brain regions that regulate emotion, psychomotor function, and reward [8]. With respect to psychological distress, studies have investigated associations between inflammatory markers and psychological distress in adults, and reported cross-sectional associations with C-reactive protein (CRP), in large population studies (Wium-Andersen et al., 2013; Baek et al., 2019), and prospective associations with Interleukin 6 (IL-6; Virtanen et al., 2015).

There are few studies of inflammatory markers in relation to psychological distress and mental disorders (depression and anxiety) during adolescence. In a recent systematic review including studies from both children and adolescents with clinical depression, five studies were found eligible for meta-analysis (D’Acunto et al., 2019). On average, individuals with clinical depression showed a non-significant trend for higher Tumor necrosis factor alpha (TNF-α) levels compared to controls. No other inflammatory markers differed between the groups. However, no conclusions should be drawn based on only five studies with small sample sizes (*n* = 17–31). In contrast, another recent systematic review which included 22 studies on children and adolescents (Colasanto et al., 2020), reported both cross-sectional and prospective associations between CRP, IL-6, and clinical depression. However, studies are generally hampered by lack of information on potential confounding factors or effect modifiers, that is, sex, obesity, physical activity, and sleep. CRP has been found to be associated with depression in obese men only (Ladwig et al., 2003). Body fat measured *via* the proxy BMI has indeed been associated with psychological distress during adolescence (Kubzansky et al., 2012). Physical activity has been reported to protect against the effect of IL-6 on depressive symptoms in primary care patients (Rethorst et al., 2011), and mild sleep disturbance has been found to moderate the associations between inflammatory markers (IL-6 and TNF-α) and depressed mood in young females (Cho et al., 2016). Additionally, some studies have investigated associations between inflammatory markers and clinical anxiety in adolescents, where CRP was reported to be cross-sectionally associated with anxiety (Copeland et al., 2012b; Khandaker et al., 2016), yet not prospectively (Copeland et al., 2012b).

C-reactive protein has been associated with depressive symptoms among adolescents in cross-sectional studies (Guan et al., 2016; Tabatabaeizadeh et al., 2018). Prospectively, inflammatory markers [transforming growth factor alpha (TGF-α), CRP, and TNF-α] have been found to predict depressive symptoms in adolescents (Moriarity et al., 2018, 2019; Walss-Bass et al., 2018). However, in the latter associations between inflammatory markers and depressive symptoms have been found to be dependent upon sex and time to follow-up, with the strongest associations when time to follow-up was above 13 months (Moriarity et al., 2018). On the other hand, null-findings have also been reported, such as non-significant prospective associations between CRP, IL-6, TNF-α, and depressive symptoms (Copeland et al., 2012a; Moriarity et al., 2018, 2019; Walss-Bass et al., 2018). As it pertains to prospective associations between inflammatory markers and anxiety symptoms during adolescence, a study investigated 39 inflammatory markers (including TGF-α, IL-6, and TNF-α) without any significant findings (Walss-Bass et al., 2018). Psychological distress is commonly used to screen mental health in healthy populations. However, few studies have explored prospective associations between inflammatory markers and psychological distress during adolescence. Increased knowledge about this association is important for prevention of psychological distress.

To summarize, limited research has examined prospective associations between inflammatory markers and psychological distress in a general adolescent population, including information on potential confounders or effect modifiers. Literature indicates a need for sex stratification when examining such associations. Thus, this study aims to test whether circulating levels of five inflammatory markers at baseline are associated with prevalence of psychological distress at follow-up 2 years later, separately for girls and boys.

## Materials and Methods

### Study Population and Design

**Figure 1:**
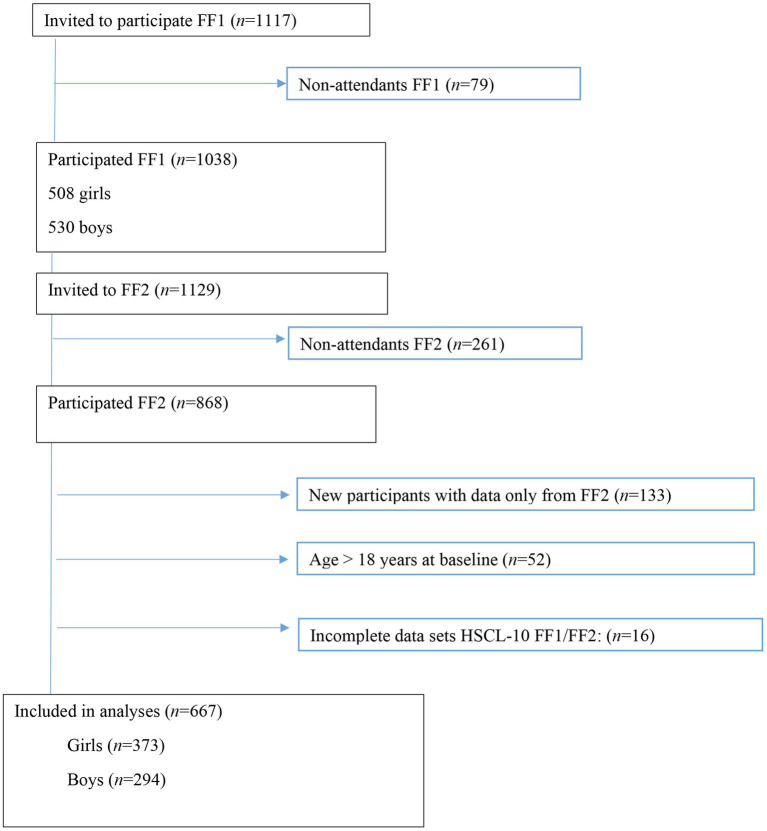
Study flowchart, Fit Futures (FF1) 2010–2011 and Fit Futures 2 (FF2) 2012–2013. HSCL-10, Hopkins Symptom Checklist.

In 2010–2011, all first-year upper secondary school students in two municipalities in Northern Norway were invited to participate in a broad health study, the Fit Futures Study. Participants were invited to a follow-up in 2012–2013. Fit Futures is thoroughly described elsewhere (Winther et al., 2014). Briefly, data were collected at the Clinical Research Unit, at the University Hospital of North Norway, Tromsø (UNN). At baseline in 2010, 1,117 students from first year in upper secondary school were invited to participate, of which 1,038 (92.9%) attended the study (FF1). In 2012–2013, all participants in FF1 and all third-year upper secondary school students were invited to participate in the follow-up study (FF2), and 868 attained. The final sample consisted of 667 participants with complete data on the outcome variable Hopkins Symptom Check List (HSCL-10) at both time-points. Participants lost to follow-up did not differ on baseline data for the inflammatory markers and HSCL-10 items (data not shown; see Figure 1).

All participants provided informed consent. Participants below 16 years additionally provided written informed consent from a parent/guardian. The study was conducted in accordance with the Declaration of Helsinki and was approved by the Norwegian Data Protection Authority (reference number 2009/1282). The Regional Committee of Medical and Health Research Ethics has also approved the study (reference number 2011/1702/REK Nord), and the present project (reference number: 2019/60811/REK Nord).

### Measurements and Questionnaires

Data about lifestyle, health and disease was collected with a web-based battery of questionnaires. Qualified research nurses conducted clinical examinations and collected blood samples. They also did interviews on medication, use of hormonal contraceptives, and acute and chronic diseases.

#### Outcome: Hopkins Symptom Check List

We used HSCL-10 to measure psychological distress. HSCL-10 was a part of the web-based questionnaire at both time-points. HSCL-10 has been found to have good psychometric properties, with both high reliability and validity (Strand et al., 2003). The scale contains four items measuring symptoms of anxiety and six items measuring symptoms of depression during the last week (Strand et al., 2003). Symptoms are reported as “none” (1), “slightly” (2), “much” (3), and “very much” (4). Cronbach’s alpha at baseline and follow-up were 0.87 and 0.90 for girls, and 0.82 and 0.87 for boys. The individual mean score of the 10 items was calculated. There were 373 girls and 294 boys with complete data on HSCL-10 at both time-points.

#### Main Exposure Variable: Pro-inflammatory Markers

Non-fasting blood samples were collected from the antecubital veins of the participants at baseline. Serum was separated and stored at −70°C. Protein extension array technology (Proseek Multiplex Inflammation panel; Olink Bioscience, Uppsala, Sweden) was used to analyze serum levels of inflammatory proteins. Details about the analysis are available elsewhere (Schistad et al., 2020). Based on previous research, the following inflammatory markers were further explored in the statistical analyses: CRP, IL-6, TGF-α, TNF-α variant 1 (TRANCE), and TNF-α variant 2 (TWEAK; Mills et al., 2013; Haapakoski et al., 2015; Walss-Bass et al., 2018). For CRP, there were 330 girls and 266 boys with data. For the four remaining inflammatory markers, there were 331 girls and 279 boys with data.

#### Baseline Covariates

Smoking and snuff use were answered by three alternatives: “daily,” “sometimes,” and “never.” Because there were few participants who smoked daily and few participants that snuffed sometimes, smoking and snuff use were recoded into dichotomous variables, with never coded as zero, and “sometimes” and “yes” were combined and both coded as one.

Alcohol consumption was measured in frequency from “never” (1) to “four or more times per week” (5). Alcohol consumption was recoded into three categories: “never,” “once per month or less,” and “twice or more per month.”

Physical activity was measured by Saltin–Grimby physical activity level scale. The scale measures physical activity during leisure time and includes type of activity and intensity during an average week in the last year (Grimby et al., 2015). The four categories were: “reading, watching TV, or other sedentary activity” (1), “walking, cycling or exercises at least 4 h a week” (2), “participation in recreational sports, heavy outdoor activities, snow clearing, etc. at least 4 h a week” (3), and “participation in hard training or sports competitions several times each week” (4). A dichotomous variable was created, with sedentary activities (the first category) coded as zero and the three other categories coded as one.

In girls, early or late menarche was measured with one question: “When did you have your first menstruation?.” From this, it was created a dichotomous (“early” vs. “late”) menarche variable based on the mean of 12.68 years of age. Self-reported menarche has good reliability (Koo and Rohan, 1997). In boys, Pubertal Development Scale (PDS; Petersen et al., 1988; Koo and Rohan, 1997) was used to measure pubertal status. The scale consisted of four questions about growth spurt, pubic hair growth, changes in voice, and facial hair growth. The four alternatives were: “have not begun” (1), “barely started” (2), 3 “underway” (3), and “completed” (4). An individual mean score was created. Further, the mean score was categorized into four categories of pubertal development: “not begun” (mean score below 2) “barely started” (mean score from 2 < 3), “underway” (mean score from 3 < 4) and “completed” (mean score of 4).

Sleep duration was measured with one question: “How many hours sleep do you normally get per night?,” with alternatives from “four or less hours” to “12 h or more.” The lowest and highest category were coded as four and 12, respectively. Further, a dichotomous variable was created based on the median hours of sleep, 7:00 h. High school program was self-reported, with the three following alternatives: “general studies,” “sports and physical,” and “vocational.”

Height and weight were measured following standard procedures, that is, in light clothing without shoes [39]. The Jenix DS 102 stadiometer (Dong Sahn Jenix, Seoul, Korea), an automatic electronic scale, was used to measure weight. Dual X-ray absorptiometry was used to measure total body fat mass (DXA; GE Lunar prodigy, Lunar Corporation, Madison, WI, United States). Body fat percentage was calculated from total fat mass in kilogram divided by weight in kilogram. Dichotomous variables for each sex were created, with cutoffs for body fat percentages of 30 and 25% in girls and boys, respectively (Marques-Vidal et al., 2008). For current infection, chronic disease and current use of hormonal contraceptives dichotomous variables were created. Self-reported medication for daily or regular use were ATC-coded (Anatomical Therapeutic Chemical code). A dichotomized variable (medication intake) was created.

Vitamin D status was measured by serum 25-hydroxyvitamin D (25(OH)D), analyzed by high pressure liquid chromatography-mass spectroscopy (LC-MS/MS) in sera stored at −80°C at Haukeland University Hospital, Norway (Grimnes et al., 2010). Stored samples were re-analyzed at the Cork Centre for Vitamin D and Nutrition Research, Ireland (Cashman et al., 2016) to be able to standardize the results according to the Vitamin D Standardization program (VSDP). More details are elaborated elsewhere (Teigmo et al., 2018). The standardized version of 25(OH)D (nmol/L) was used as a continuous variable.

Self-rated health was reported with one question: “How do you in general consider your own health to be?” The five categories were: “very bad” (1), “bad” (2), “neither bad nor god” (3), “good” (4), and “excellent” (5). This question has been found to predict mortality (Idler and Benyamini, 1997), and morbidity in the general population (Latham and Peek, 2013). Further, a change score was calculated between follow-up and baseline so that positive and negative scores indicated better and worse self-rated health condition at follow-up versus at baseline, respectively.

### Statistical Analysis

All statistical analyses were conducted with the Statistical Package of Social Science (SPSS v. 26). A significance level of *p* < 0.05 as an indication of statistical significance was chosen. We excluded participants aged 19 years or older at baseline, and with incomplete data on HSCL-10 at baseline and/or follow-up (Figure 1). Residual analysis was conducted to assess linearity, distribution, variance homogeneity, and to detect outliers. Exposure variables and potential confounders were tested for multicollinearity.

Because girls exhibit higher levels of depressive symptoms than boys in adolescence (Bakken, 2019) and because the associations between inflammatory markers and depressive symptoms have been sex-dependent in former studies (Moriarity et al., 2018), all analyses were done separately for girls and boys. Paired-samples *t*-tests were conducted to compare HSCL-10 at baseline and HSCL-10 at follow-up. Independent samples *t*-tests were conducted to compare HSCL-10 levels between girls and boys at baseline and at follow-up, respectively.

Variables assessed at baseline are presented for girls and boys separately, with means and standard deviations for continuous variables with normal distribution, median and IQR for continuous variables with skewness, and proportions for categorical variables. Data from girls and boys were compared in the following way: continuous data with normal distribution with independent sample *t*-tests, continuous data with skewness with Mann–Whitney *U*-tests, and categorical data with chi-square tests.

Linear regressions were conducted to estimate the unstandardized beta regression coefficients and 95% confidence intervals (CI) between pro-inflammatory markers at baseline and psychological distress at follow-up (the latter serving as outcome variable). Firstly, crude associations between the respective inflammatory markers and psychological distress were estimated (Model 1). Secondly, adjustment for baseline psychological distress was conducted (Model 2). Thirdly, potential confounders were added (Model 3). Potential confounders, coded as presented in Table 1 were first tested in simple linear regressions with the mean of HSCL-10 score at follow-up as outcome and were included in the multivariable regression analysis (Model 3) when the value of *p* was below 0.10. For self-rated health, both the baseline score and the change score (follow-up minus baseline) were added simultaneously. This was done to adjust for baseline self-rated health in addition to change in self-rated health from FF1 to FF2 (Supplementary Table 1).

**Table 1:** Characteristics at baseline for girls and boys. Fit Futures 2010-2011.

	Girls		Boys		*p*-Value^*^
	*n*	Mean (SD)	*n*	Mean (SD)	
Age in years, mean (SD)	373	16.15 (0.44)	294	16.11 (0.52)	0.230
Self-rated health	370	3.93 (0.76)	293	3.94 (0.87)	0.923
Body fat percentage, dichotomous^a^	373		294		N/A
<Cutoff	159	42.6%	215	73.1%	
≥Cutoff	214	57.4%	79	26.9%	
Age menarche (years)	367				
Early (≤12.70)	141	38.4%			
Late (>12.70)	226	61.6%			
PDS status			239		
Completed			23	9.6%	
Underway			172	72.0%	
Barely started			44	18.4%	
Not begun				0%	
Smoking	373		294		0.771
No, never	304	81.5%	237	80.6%	
Yes	69	18.5%	57	19.4%	
Snuffing	373		293		0.407
No, never	257	68.9%	193	65.9%	
Yes	116	31.1%	100	34.1%	
Alcohol	373		293		**0.039**
Never	95	25.5%	101	34.5%	
Once per month or less	172	46.1%	116	39.6%	
Twice or more per month	106	28.4%	76	25.9%	
Physical activity	373		294		**<0.001**
Sedentary	47	12.6%	82	27.9%	
Active	326	87.4%	212	72.1%	
Sleep duration (hours)^b^	373		291		0.793
<Cutoff	146	39.1%	111	38.1%	
≥Cutoff	227	60.9%	180	61.9%	
Current infection	371		293		0.387
No	318	85.7%	244	83.3%	
Yes	58	14.3%	49	16.7%	
Chronic disease	371		292		0.247
No	254	68.5%	212	72.6%	
Yes	117	31.5%	80	27.4%	
Hormonal contraceptives	370				
No	229	61.9%			
Yes	141	38.1%			
Intake of medication	371		293		**<0.001**
No	257	69.3%	239	81.6%	
Yes	114	30.7%	54	18.4%	
High school program	373		294		**<0.001**
General studies	203	54.4%	107	36.4%	
Sports and physical	35	9.4%	43	14.6%	
Vocational	135	36.2%	144	49.0%	
		Median (IQR)		Median (IQR)	
CRP, mg/L Median and IQR	330	0.54 (1.11)	266	0.53 (0.87)	0.542
IL-6, NPX Median and IQR	331	2.70 (0.58)	279	2.72 (0.61)	0.994
TGF-α, NPX Median and IQR	331	3.89 (0.75)	279	3.61 (0.70)	**<0.001**
TRANCE, NPX Median and IQR	331	5.54 (0.79)	279	6.03 (0.70)	**<0.001**
TWEAK, NPX Median and IQR	331	8.88 (0.44)	279	9.02 (0.37)	**<0.001**
25(OH)D, nmol/L Median and IQR	331	41.0 (24.6)	280	30.9 (21.3)	**<0.001**

Mean (SD) of continuous variable and percentages of categorical variables are reported. Median and IQR are reported for inflammatory markers and Vitamin D. PDS, Pubertal Development Scale; Intake of medication, Intake of medications, analgesics, or antibiotics in the last 24h; CRP, C-reactive protein; IL6-α, Interleukin 6 alpha; TGF-α, Transforming growth factor alpha; TRANCE, Tumor Necrosis Factor-related activation-induced cytokine (O14788: TNF-related activation-induced cytokine within limits of detection); TWEAK, Tumor necrosis factor-like weak inducer of apoptosis (O43508: TNF-like weak inducer of apoptosis within limits of detection); NPX, Normalized protein expression; 25(OH)D, Standardized version of (25-OH)D; N/A, Not applicable. Bold, Statistically significant with a value of p of 0.05.

a Cutoff body fat percentage girls (30) and boys (25).

b Cutoff sleep duration 7.00h.

* Chi-square for categorical variables and t-test or Mann–Whitney U for continuous variables.

As supplementary analysis, we did linear regressions with the mean score of the six items of depressive symptoms from HSCL-10 at follow-up as outcome. This was done to assess the association between depressive symptoms and inflammatory markers more directly, without confounding the findings with the severity of anxiety.

## Results

### Characteristics at Baseline and Outcome Distribution

Characteristics at baseline for girls and boys are presented in Table 1. In girls and boys, 57.4 and 26.9%, respectively, were measured above the sex-specific cutoffs for body fat percentage of 30 and 25. Sedentary behavior was more prevalent in boys (27.9%) than in girls (12.6%). A higher proportion of girls (30.7%) used medication compared to boys (18.4%). Among girls, the most common high school program was general studies (54.4%), while among boys, vocational studies were most common (49.0%). Drinking alcohol was more common in girls than in boys. TGF-α and 25(OH)D were significantly higher in girls than in boys, whereas TRANCE and TWEAK were lower (Table 1).

**Figure 2:**
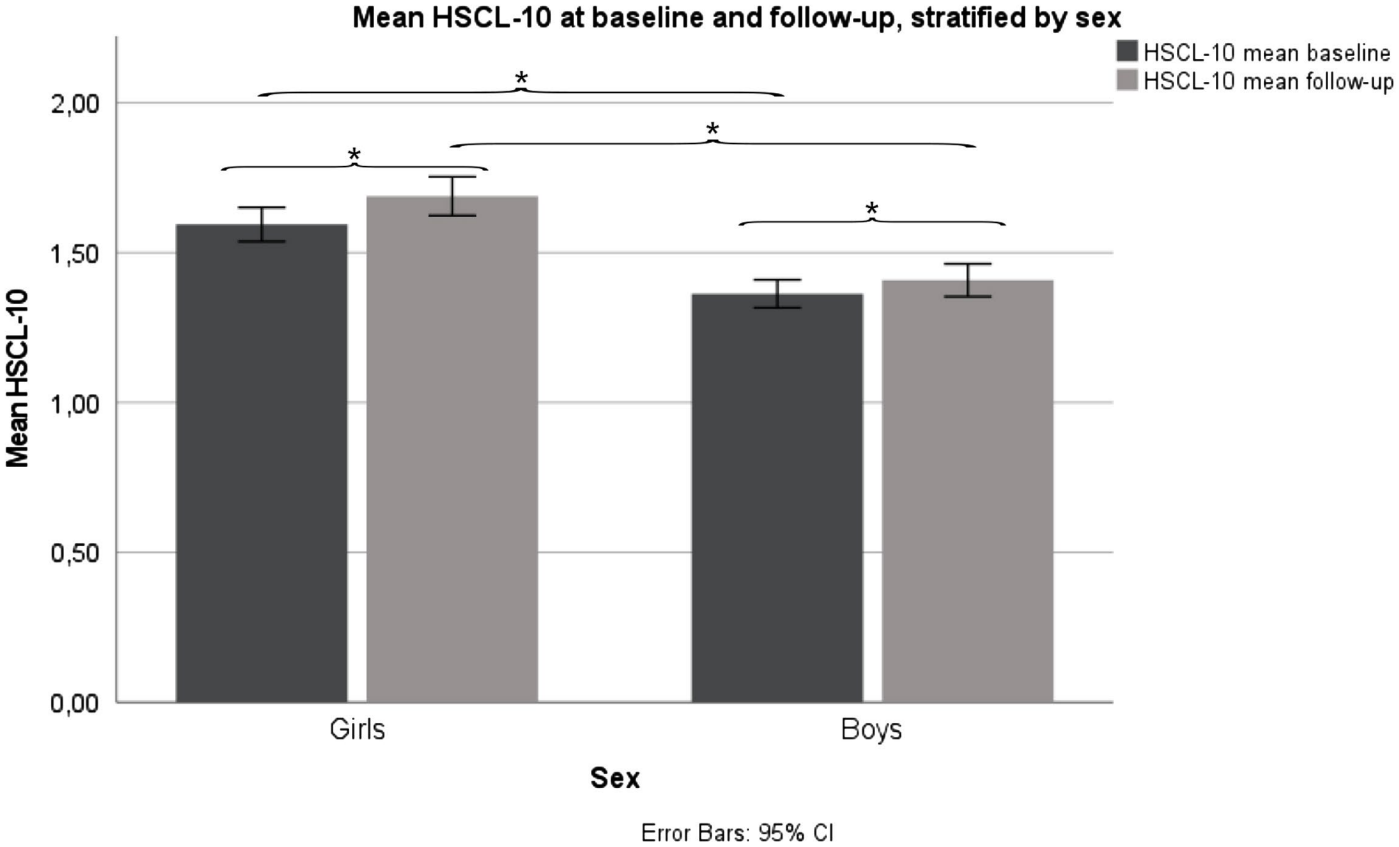
Mean HSCL-10 levels, stratified by sex. Fit Futures (FF1) 2010–2011 and Fit Futures 2 (FF2) 2012–2013. ^*^p < 0.001. HSCL-10, Hopkins Symptom Checklist.

Girls reported a mean (SD) HSCL-10 of 1.59 (0.56) at baseline and 1.69 (0.64) at follow-up, a statistically significant increase in HSCL-10 from baseline to follow-up, *p* < 0.001. Girls scored significantly higher compared to boys at baseline, *p* < 0.001, and follow-up, *p* < 0.001. Boys reported a mean (SD) HSCL-10 of 1.36 (0.41) at baseline, and 1.41 (0.48) at follow-up. Also, in boys, there was a statistically significant increase in HSCL-10 from baseline to follow-up, *p* < 0.001 (Figure 2).

### Prospective Associations Between Inflammatory Markers and Psychological Distress

In girls, all inflammatory markers were positively associated with HSCL-10 in crude analyses, except for TRANCE, which showed a negative association. However, none of these associations were statistically significant (Table 2). None of the adjusted analyses (models 2 and 3) showed statistically significant associations. In boys, both CRP [*b* = 0.021, 95% CI (0.003 and 0.039)] and TGF-α [*b* = 0.134, 95% CI (0.037 and 0.230)] at baseline showed significant associations with HSCL-10 at follow-up. Both associations remained statistically significant after adjustments (Table 2).

**Table 2:** Crude and adjusted associations between baseline inflammatory proteins and HSCL-10 at follow-up, assessed by linear regressions.

	*n*	*B*	95% CI		*p*-Value	*R*^2^ change
			Lower	Upper		
Girls						
CRP						
Model 1	330	0.017	−0.004	0.039	0.118	0.008
Model 2	330	0.013	−0.005	0.030	0.152	**0.363**
Model 3	324	0.005	−0.013	0.023	0.575	**0.057**
IL-6						
Model 1	331	0.064	−0.044	0.171	0.247	0.004
Model 2	331	0.035	−0.051	0.122	0.419	**0.362**
Model 3	325	0.002	−0.086	0.090	0.964	**0.059**
TGF-α						
Model 1	331	0.046	−0.070	0.161	0.437	0.002
Model 2	331	0.057	−0.035	0.150	0.221	**0.366**
Model 3	325	0.039	−0.051	0.128	0.397	**0.059**
TRANCE (TNF)						
Model 1	331	−0.016	−0.129	0.097	0.776	<0.001
Model 2	331	0.039	−0.051	0.130	0.393	**0.366**
Model 3	325	0.006	−0.086	0.098	0.895	**0.059**
TWEAK (TNF)						
Model 1	331	0.058	−0.153	0.270	0.588	0.001
Model 2	331	0.108	−0.061	0.276	0.209	**0.367**
Model 3	325	0.123	−0.047	0.293	0.155	**0.061**
Boys						
CRP						
Model 1	266	0.021	0.003	0.039	0.022^*^	**0.020**
Model 2	266	0.027	0.011	0.043	0.001^*^	**0.255**
Model 3	262	0.026	0.010	0.042	0.002^*^	0.011
IL-6						
Model 1	279	0.035	−0.054	0.124	0.443	0.002
Model 2	279	0.062	−0.013	0.138	0.105	**0.286**
Model 3	274	0.064	−0.011	0.139	0.096	0.016
TGF-α						
Model 1	279	0.134	0.037	0.230	0.004^*^	**0.030**
Model 2	279	0.123	0.042	0.203	0.003^*^	**0.274**
Model 3	274	0.122	0.040	0.203	0.004^*^	0.014
TRANCE (TNF)						
Model 1	279	−0.022	−0.130	0.086	0.688	0.001
Model 2	279	−0.004	−0.096	0.087	0.929	**0.281**
Model 3	274	0.001	−0.091	0.093	0.982	0.015
TWEAK (TNF)						
Model 1	279	0.012	−0.189	0.214	0.903	<0.001
Model 2	279	0.039	−0.132	0.210	0.652	**0.282**
Model 3	274	0.042	−0.129	0.212	0.631	0.015

The results are presented for girls and boys, respectively. Fit futures 2010–2011 and 2012–2013. B, Unstandardized beta; CRP, C-reactive protein; IL6-α, Interleukin 6 alpha; TGF-α, Transforming growth factor alpha; TRANCE, Tumor Necrosis Factor-related activation-induced cytokine (O14788: TNF-related activation-induced cytokine within limits of detection); TWEAK, Tumor necrosis factor-like weak inducer of apoptosis (O43508: TNF-like weak inducer of apoptosis within limits of detection); Model 1, Crude analysis with the respective inflammatory marker; Model 2, Model 1+ baseline HSCL-10; Model 3 girls, Model 2+ current infection, medication intake, sleep duration, hormonal contraceptives, smoking, self-rated health, and change score self-rated health (inclusion criteria 0.1 from simple regressions); Model 3 boys, Model 2+ physical activity, sleep duration, self-rated health, and change score self-rated health (inclusion criteria 0.1 from simple regressions). Bold, Significant *R*^2^ change.

* Statistically significant with a value of *p* of 0.05.

### Effect Modification by Body Fat Percentage, Physical Activity, and Sleep

Interaction terms were tested to examine if associations between baseline inflammatory markers and psychological distress at follow-up differed by dichotomous versions of physical activity levels, body fat percentage, and sleep duration. Interaction terms were tested in simple linear regressions and included in further analysis when the value of *p* was below 0.05. In girls, there was a significant interaction between CRP and sleep duration (*p* = 0.007). In boys, there were significant interactions between CRP and both body fat percentage (*p* = 0.009) and physical activity (*p* = 0.025). Further, in boys, there were significant interactions between TGF-α and body fat percentage (*p* = 0.028), TRANCE and physical activity (*p* = 0.037), and TWEAK and sleep duration (*p* = 0.038). The significant interaction terms concerning these covariates were added to the fully adjusted models for the respective inflammatory markers. For girls, an interaction term between CRP and sleep duration was added to the fully adjusted model. For boys, interaction terms between CRP and body fat percentage and physical activity were added separately in two models. Further, in boys, interaction terms between TGF-α and body fat percentage, TRANCE and physical activity, and TWEAK and sleep duration were added in the three respective models. The interaction terms were interpreted as statistically significant when their value of *p* was below 0.05, and when there was a significant increase in *R*^2^ change from the respective versions of Model 3.

In boys, body fat percentage [*b* = 0.042, 95% CI (0.008 and 0.075)] and physical activity [*b* = −0.045, 95% CI (−0.076 and −0.013)] moderated the association between CRP and HSCL-10. Sleep duration [*b* = −0.046, 95% CI (−0.750 and −0.062)] moderated the association between TWEAK and HSCL-10. The significant interaction terms were further investigated with stratified analyses. The results showed a positive association between CRP and HSCL-10 in boys with body fat percentage ≥25 [*b* = 0.041, 95% CI (0.013 and 0.069)] and in sedentary boys [*b* = 0.049, 95% CI (0.019 and 0.080)], and that TWEAK predicted HSCL-10 in boys that slept ≥7 h per night [*b* = 0.212, 95% CI (0.011 and 0.412); Table 3]. For more details about winning models, see Supplementary Table 2.

**Table 3:** Stratified analyses of the associations between baseline inflammatory proteins and HSCL-10 at follow-up, assessed by linear regressions.

	*n*	*B*	95% CI		*p*-Value
			Lower	Upper	
Boys					
CRP Model A					
<25% body fat	195	0.002	−0.022	0.026	0.877
≥25% body fat	67	0.041	0.013	0.069	0.005^*^
CRP Model B					
Sedentary	68	0.049	0.019	0.080	0.002^*^
Active	194	0.006	−0.014	0.025	0.573
TWEAK Model					
<7.00 h sleep duration	101	−0.188	−0.501	0.124	0.234
≥7.00 h sleep duration	173	0.212	0.011	0.412	0.038^*^

Boys in Fit futures 2010–2011 and 2012–2013. B, Unstandardized beta; CRP, C-reactive protein; TWEAK, Tumor necrosis factor-like weak inducer of apoptosis (O43508: TNF-like weak inducer of apoptosis within limits of detection); CRP Model A, CRP, baseline HSCL-10, physical activity, sleep duration, self-rated health, and change score self-rated health; CRP Model B, CRP, baseline HSCL-10, sleep duration, self-rated health, and change score self-rated health; TWEAK Model, TWEAK, baseline HSCL-10, physical activity, self-rated health, and change score self-rated health.

* Statistically significant with a value of *p* of 0.05.

### Supplementary Analysis Using the Six Items Depressive Symptoms as Outcome

Supplementary analysis restricted to the six HSCL-10 items measuring depressive symptoms as outcome and covariate did not alter the significant associations with CRP and TGF-α in boys (Supplementary Table 3).

## Discussion

In this population-based longitudinal study among adolescents, boys with higher levels of CRP and TGF-α at baseline reported more psychological distress at two-year follow-up. The findings remained significant after adjustment for confounding factors. The associations were not found in girls. Levels of TRANCE, TWEAK, and IL-6 were not associated with psychological distress.

In boys, we found a prospective association between CRP at baseline and psychological distress at follow-up where an increase of 0.01 mg/L in CRP indicated an increase of 0.021 units in HSCL-10 at follow-up. The positive association is in line with findings from Moriarity et al. (2018). Their study was conducted in a community sample (*n* = 201, 109 females) aged 12.3–20 years, with a mean age of 16.8 years at first blood draw (Moriarity et al., 2018). Moriarity et al. found a small effect from CRP on depressive symptoms measured by Children’s Depression Inventory (CDI). In contrast, we found a positive effect of CRP only in boys. At the same time, girls in our sample had higher levels of psychological distress at baseline and follow-up, yet we found no differences in CRP levels between girls and boys at baseline. Regardless of the differences between our study and the study of Moriarity et al. (2018), both studies indicate that CRP is positively associated with the respective outcomes; depressive symptoms and psychological distress.

Most studies among healthy adolescents, have not found significant prospective associations between CRP and depressive symptoms (Miller and Cole, 2012; Copeland et al., 2012a; Khandaker et al., 2014; Khandaker et al., 2018; Moriarity et al., 2019). A study by Moriarity et al. (2019) found no association between CRP and depressive symptoms measured with CDI. This study included 140 adolescents (54% girls) with a mean age of 16.1 years. Our study resembles this sample as it pertains to age, health of participants, and time to follow-up above 13 months as recommended by Moriarity et al. (2018). A crucial difference is that we stratified on sex. To our knowledge, our study is the first study to find an effect of CRP on psychological distress across adolescence among healthy boys. Clearly, more studies are warranted to explore the association between CRP and psychological distress during adolescence and investigate potential sex differences.

We found that higher levels of TGF-α at baseline increased psychological distress 2 years later in boys. To our knowledge, this is the first study to find an effect of TGF-α on psychological distress during adolescence. Our result corresponds with a study from Walss-Bass et al. (2018) indicating that TGF-α predicted depressive symptoms measured with Mood-Feelings Questionnaire-Child (MFQC), in a sample of 254 adolescents aged 12–15 years (54% female at baseline), with follow-ups 1 and 2 years later.

Contrary to the study by Walss-Bass et al., our study found associations between TGF-α and outcome in boys only. In our sample, girls had significantly higher levels of TGF-α at baseline (*p* < 0.01) compared to boys. We are not aware of other studies, except from the aforementioned (Walss-Bass et al., 2018) that have investigated prospective associations between TGF-α and depressive symptoms or psychological distress during adolescence. Thus, the finding in our study warrants further study of the prospective associations between TGF-α and psychological distress during adolescence, including investigations of sex differences.

The null-findings from the present study for IL-6, TRANCE, and TWEAK are in line with previous findings showing that inflammatory markers are more strongly prospectively associated with clinical depression than with depressive symptoms. Indeed, in a cumulative meta-analysis in adult patients with clinical depression, conducted on 58 observational studies, it was concluded that IL-6, CRP, and TNF-α were more strongly associated with severe depression than with moderate depression (Haapakoski et al., 2015). Because the associations between inflammatory markers and depression get stronger with more severe depression, weaker and more inconsistent findings are expected when using depressive symptoms. It may also be that TGF-α is only associated with psychological distress and depressive symptoms, whereas IL-6 and TNF-α are more sensitive predictors of clinical depression, and that CRP is a sensitive predictor for both clinical depression and depressive symptoms/psychological distress.

The inconsistent findings across studies may be explained by use of different outcomes. In our study, we used HSCL-10, while other studies have used measures, such as CDI and MFQC. The respective measurements of depressive symptoms aim to measure the same latent construct and are all validated and reliable measures of depressive symptoms. On the other hand, Moriarity et al. (2018) reported that many of their participants were nearing the upper end of the age range for which CDI has been validated. Different measurement scales of depressive symptoms may vary in what kind of symptoms their items are covering. Indeed, some studies have reported that inflammatory markers are associated with only specific depressive symptoms (Moriarity et al., 2018). Some scales might have more items about dysphoria and anhedonia, while other include more items about bodily symptoms, such as fatigue, pain, and sleeplessness. Whether there is an association between inflammatory markers and total depressive symptoms may be dependent on which symptoms that are included and weighted in the respective measurement scale.

In boys, there were statistically significant interactions between CRP, body fat percentage, and physical activity. This indicates that the effect of CRP on psychological distress is dependent upon body fat percentage and physical activity. These interaction effects are in line with studies on adolescents showing that body fat percentage and less physical activity are associated with increased systemic inflammation (Platat et al., 2006; Warnberg et al., 2007).

The significant interaction for body fat percentage and physical activity indicates that CRP has stronger association with psychological distress at 2 years of follow-up (after controlling for confounders, most importantly, for distress at baseline, and for self-assessed health condition) in boys that have high body fat percentage and/or are less physically active. One might speculate that low body fat percentage and physical activity among boys protects against the deleterious effects of CRP-associated inflammation on psychological distress in the long-run. Alternatively, high body fat percentage and physical inactivity, respectively, may increase inflammation which in the next step increases psychological distress. Otherwise, it may be that inflammation *per se* is not the driving factor for increased psychological distress, but rather a marker of unhealthy lifestyle (such as high body fat and low levels of physical activity) that are the actual drivers. Regardless of the mechanisms, the results in our study suggest that interventions should be designed to promote lower body fat percentage and more physical activity in boys to prevent increases in inflammation and psychological distress.

In boys, we also found a statistically significant interaction between TWEAK and sleep, indicating that the prospective association between TWEAK and psychological distress is dependent upon sleep duration. Surprisingly, an increase in TWEAK predicted increased psychological distress in boys that slept ≥7 h. This contrasts a study by Cho et al. (2016) that found stronger correlations between inflammatory markers (IL-6 and TNF-α) and depressed mood in young females with mild sleep disturbance compared to controls. In males, no moderating effect from sleep disturbance was found (Cho et al., 2016). Our finding may be spurious and could be explained by a median sleep duration of 7 h in our sample, which is below the recommendation of 8–10 h per night for adolescents (Paruthi et al., 2016). Nevertheless, shorter sleep has been associated with increased inflammation (Fernandez-Mendoza et al., 2017) and increased depressive symptoms (Gangwisch et al., 2010) in adolescents. Hence, further research on the association between sleep and depressive symptoms is warranted.

This study has several strengths. We explored the prospective associations between inflammatory markers and psychological distress as outcome in healthy adolescents, a population that have been understudied regarding such associations. Second, the sample size in our study was large enough to allow sex stratification. Third, the study explored several inflammatory markers that previously have been found to be prospectively associated with depressive symptoms during adolescence. This is in line with a call for a novel exploration of different markers (Mills et al., 2013). Finally, the present study examined several confounders recommended in literature (Haapakoski et al., 2015).

This study has some limitations. We used self-reported psychological distress as outcome. Inflammatory markers have been reported to show stronger associations with depressive symptoms measured through clinical interviews than in self-report. For CRP, the effect size has been reported to be twice as strong when using structured interviews compared to questionnaires (Howren et al., 2009). Nevertheless, HSCL-10 is considered a reliable and valid instrument for measurement of psychological distress (Finbråten et al., 2021). Secondly, because the prospective associations between inflammatory markers and psychological distress are relatively weak, the sample size may have been too small to detect associations. Large population studies that have detected statistically significant associations between CRP and psychological distress in adults used sample sizes of approximately 70,000 participants (Wium-Andersen et al., 2013; Baek et al., 2019). On the other hand, weak associations may not be clinically relevant. Thus, smaller sample sizes, as the one applied in our study, are justified. Although we found statistically significant associations in our study, the changes in HSCL-10 were small. It is unclear whether these associations are clinically significant. Future studies should examine the clinical significance of such associations.

According to our study, increased levels of CRP and TGF-α are associated with the development of more psychological distress across adolescence in boys. Other inflammatory markers that have been associated with clinical depression or anxiety (IL-6, TRANCE, and TWEAK) seem to be less relevant for psychological distress in adolescence. Our study suggests that boys who are physically inactive or have a higher body fat mass are particularly vulnerable to higher CRP, while surprisingly, boys who sleep longer are more vulnerable to higher TWEAK levels. Thus, investigation of the moderating role of body fat percentage, physical activity, and sleep duration are warranted in future prospective studies examining associations between inflammatory markers and psychological distress during adolescence.

## Data Availability Statement

The data analyzed in this study is subject to the following licenses/restrictions: Data may be obtained from a third party, UiT—The Artic University of Norway. Restrictions apply to the availability of these data, which were used under license for the current study, and are thus not publicly available. Requests to access these datasets should be directed to Elin Kristin Evensen, elin.k.evensen@uit.no.

## Ethics Statement

The Regional Committee of Medical and Health Research Ethics has also approved the study (reference number 2011/1702/REK Nord), and the present project (reference number: 2019/60811/REK Nord). Written informed consent to participate in this study was provided by the participants’ legal guardian/next of kin.

## Author Contributions

NE, A-SF, GG, and LA contributed to the study conception and design. Material preparation, data collection, and analysis were performed by JL, TC, LA, and GC. The first draft of the manuscript was written by JL. All authors contributed to the article and approved the submitted version.

## Funding

The research project is financed through Department of Health and Care Sciences, UiT Arctic University of Norway.

## Conflict of Interest

The authors declare that the research was conducted in the absence of any commercial or financial relationships that could be construed as a potential conflict of interest.

## Publisher’s Note

All claims expressed in this article are solely those of the authors and do not necessarily represent those of their affiliated organizations, or those of the publisher, the editors and the reviewers. Any product that may be evaluated in this article, or claim that may be made by its manufacturer, is not guaranteed or endorsed by the publisher.
