# Corrigendum: C-Reactive Protein and TGF-α Predict Psychological Distress at Two Years of Follow-Up in Healthy Adolescent Boys: The Fit Futures Study

**DOI:** 10.3389/fpsyg.2022.953498

**Published:** 2022-07-01

**Authors:** Jonas Linkas, Luai Awad Ahmed, Gabor Csifcsak, Nina Emaus, Anne-Sofie Furberg, Guri Grimnes, Gunn Pettersen, Kamilla Rognmo, Tore Christoffersen

**Affiliations:** ^1^Department of Health and Care Sciences, UiT The Arctic University of Norway, Narvik, Norway; ^2^Institute of Public Health, College of Medicine and Health Sciences, United Arab Emirates University, Al Ain, United Arab Emirates; ^3^Department of Psychology, UiT The Arctic University of Norway, Tromsø, Norway; ^4^Department of Health and Care Sciences, UiT The Arctic University of Norway, Tromsø, Norway; ^5^Faculty of Health and Care Sciences, Molde University College, Molde, Norway; ^6^Division of Internal Medicine, University Hospital of North Norway, Tromsø, Norway; ^7^Institute of Clinical Medicine, UiT The Arctic University of Norway, Tromsø, Norway; ^8^School of Sport Sciences, UiT The Arctic University of Norway, Alta, Norway; ^9^Department of Research and Development, Finnmark Hospital Trust, Alta, Norway

**Keywords:** psychological distress, inflammatory markers, depressive symptoms, anxiety symptoms, adolescence

In the original article, there was a mistake in Table 1 as published. The rows “IL-6,” “TGF-α,” “TRANCE TWEAK” were presented in pg/L. This is not the correct unit. The correct unit is NPX. The corrected Table 1 appears below and the footnote has been updated accordingly as well.

**Table 1:** Characteristics at baseline for girls and boys. Fit Futures 2010–2011.

	**Girls**		**Boys**		
	***n***	**Mean (SD)**	***n***	**Mean (SD)**	*p*-value*
Age in years Mean (SD)	373	16.15 (0.44)	294	16.11 (0.52)	0.230
Self-rated health	370	3.93 (0.76)	293	3.94 (0.87)	0.923
Body fat percentage, dichotomous^a^	373		294		N/A
< Cutoff	159	42.6%	215	73.1%	
≥Cutoff	214	57.4%	79	26.9%	
Age menarche (years)	367				
Early (≤ 12.70)	141	38.4%			
Late (>12.70)	226	61.6%			
PDS status			239		
Completed			23	9.6%	
Underway			172	72.0%	
Barely started			44	18.4%	
Not begun				0%	
Smoking	373		294		0.771
No, never	304	81.5%	237	80.6%	
Yes	69	18.5%	57	19.4%	
Snuffing	373		293		0.407
No, never	257	68.9%	193	65.9%	
Yes	116	31.1%	100	34.1%	
Alcohol	373		293		**0.039**
Never	95	25.5%	101	34.5%	
Once per month or less	172	46.1%	116	39.6%	
Twice or more per month	106	28.4%	76	25.9%	
Physical activity	373		294		**<0.001**
Sedentary	47	12.6%	82	27.9%	
Active	326	87.4%	212	72.1%	
Sleep duration (hours)^b^	373		291		0.793
< Cutoff	146	39.1%	111	38.1%	
≥Cutoff	227	60.9%	180	61.9%	
Current infection	371		293		0.387
No	318	85.7%	244	83.3%	
Yes	58	14.3%	49	16.7%	
Chronic disease	371		292		0.247
No	254	68.5%	212	72.6%	
Yes	117	31.5%	80	27.4%	
Hormonal contraceptives	370				
No	229	61.9%			
Yes	141	38.1%			
Intake of medication	371		293		**<0.001**
No	257	69.3%	239	81.6%	
Yes	114	30.7%	54	18.4%	
High school program	373		294		**<0.001**
General studies	203	54.4%	107	36.4%	
Sports and physical	35	9.4%	43	14.6%	
Vocational	135	36.2%	144	49.0%	
		Median (IQR)		Median (IQR)	
CRP mg/L Median and IQR	330	0.54 (1.11)	266	0.53 (0.87)	0.542
IL-6 NPX Median and IQR	331	2.70 (0.58)	279	2.72 (0.61)	0.994
TGF-α NPX Median and IQR	331	3.89 (0.75)	279	3.61 (0.70)	**<0.001**
TRANCE NPX Median and IQR	331	5.54 (0.79)	279	6.03 (0.70)	**<0.001**
TWEAK NPX Median and IQR	331	8.88 (0.44)	279	9.02 (0.37)	**<0.001**
25(OH)D nmol/L Median and IQR	331	41.0 (24.6)	280	30.9 (21.3)	**<0.001**

*Mean (SD) of continuous variable and percentages of categorical variables are reported. Median and IQR are reported for inflammatory markers and Vitamin D. PDS, Pubertal Development Scale; Intake of medication, Intake of medications, analgesics, or antibiotics in the last 24h; CRP, C-reactive protein; IL6-a, Interleukin 6 alpha; TGF-a, Transforming growth factor alpha; TRANCE, Tumor Necrosis Factor-related activation-induced cytokine (O14788: TNF-related activation-induced cytokine within limits of detection); TWEAK, Tumor necrosis factor-like weak inducer of apoptosis (O43508: TNF-like weak inducer of apoptosis within limits of detection); NPX, Normalized protein expression; 25(OH)D, Standardized version of (25-OH)D; N/A, Not applicable. Bold, Statistically significant with a value of p of 0.05.*

a *Cutoff body fat percentage girls (30) and boys (25).*

b *Cutoff sleep duration 7.00h. Chi-square for categorical variables and t-test or Mann–Whitney U for continuous variables*.

Additionally, the mean and standard deviation for the row “Self-rated health” in the column “Girls” were presented as “3.93 (0.64)”. This standard deviation is wrong. The correct is “(0.76)”. The corrected Table 1 appears below.

Additionally the caption of the table was incorrect. It was originally “Characteristics at baseline for girls and boys” but should be “Characteristics at baseline for girls and boys. Fit Futures 2010-2011.” The corrected Table 1 appears below.

The authors apologize for this error and state that this does not change the scientific conclusions of the article in any way. The original article has been updated.

## Publisher's Note

All claims expressed in this article are solely those of the authors and do not necessarily represent those of their affiliated organizations, or those of the publisher, the editors and the reviewers. Any product that may be evaluated in this article, or claim that may be made by its manufacturer, is not guaranteed or endorsed by the publisher.

