# Supplementary material for: C-Reactive Protein and TGF-α Predict Psychological Distress at Two Years of Follow-Up in Healthy Adolescent Boys: The Fit Futures Study
Source: Front Psychol. 2022 Mar 11;13:823420. doi: 10.3389/fpsyg.2022.823420 (PMC8963454; doi:10.3389/fpsyg.2022.823420)
Supplement: Supplementary file 1 [file Table_1.DOCX]

**Supplementary table 1:** Assessing self-rated health and change score of self-rated health as confounders. *Associations between self-rated health and the HSCL-10 at follow-up, assessed by linear regressions. The results are presented for girls and boys, respectively. Fit futures 2010-2011 and 2012-2013.*

|  |  | 95 % CI | |  |  |  |  |
| --- | --- | --- | --- | --- | --- | --- | --- |
| Girls | *B* | Lower | Upper | p-value | R square change |  |  |
| Model 1 |  |  |  |  | **0.021** |  |  |
| Change score self-rated health | -0.111 | -0.190 | -0.033 | 0.006* |  |  |  |
| Model 2 |  |  |  |  | **0.104** |  |  |
| Change score self-rated health | -0.252 | -0.337 | -0.216 | <0.001* |  |  |  |
| Self-rated health baseline | -0.308 | -0.401 | -0.216 | <0.001* |  |  |  |
| Model 3 |  |  |  |  | 0.003 |  |  |
| Change score self-rated health | -0.092 | -0.410 | 0.225 | 0.567 |  |  |  |
| Self-rated health baseline | -0.306 | -0.399 | -0.214 | <0.001* |  |  |  |
| Interaction^a^ | -0.043 | -0.124 | 0.039 | 0.305 |  |  |  |
| Boys |  |  |  |  |  |  |  |
| Model 1 |  |  |  |  | 0.001 |  |  |
| Change score self-rated health | -0.015 | -0.081 | 0.052 | 0.663 |  |  |  |
| Model 2 |  |  |  |  | **0.076** |  |  |
| Change score self-rated health | -0.109 | -0.184 | -0.035 | 0.004* |  |  |  |
| Self-rated health baseline | -0.176 | -0.247 | -0.105 | <0.001* |  |  |  |
| Model 3 |  |  |  |  | <0.001 |  |  |
| Change score self-rated health | -0.082 | -0.317 | 0.153 | 0.492 |  |  |  |
| Self-rated health baseline | -0.175 | -0.246 | -0.103 | <0.001* |  |  |  |
| Interaction^a^ | -0.007 | -0.068 | 0.053 | 0.812 |  |  |  |

*B*: Unstandardized beta

*Statistically significant with a p-value of 0.05

Bold: Significant R^2^ change

a: Change score self-rated health* self-rated health baseline
