# Supplementary material for: C-Reactive Protein and TGF-α Predict Psychological Distress at Two Years of Follow-Up in Healthy Adolescent Boys: The Fit Futures Study
Source: Front Psychol. 2022 Mar 11;13:823420. doi: 10.3389/fpsyg.2022.823420 (PMC8963454; doi:10.3389/fpsyg.2022.823420)
Supplement: Supplementary file 2 [file Table_2.DOCX]

**Supplementary table 2**: *Winning models.* *Crude and adjusted* *associations between baseline inflammatory proteins and HSCL-10 at follow-up, assessed by linear regressions. Boys in Fit futures 2010-2011 and 2012-2013.*

| Boys |  | | |  | | |  | |  |  |
| --- | --- | --- | --- | --- | --- | --- | --- | --- | --- | --- |
|  | | | | | 95 % CI | | | | |  |
|  | | *n* | *B* | | | Lower | | Upper | | *p*-value |
| CRP Model A | | 262 |  | | |  | |  | |  |
| CRP | |  | 0.001 | | | -0.025 | | 0.027 | | 0.914 |
| Baseline HSCL-10 | |  | 0.610 | | | 0.466 | | 0.753 | | <0.001* |
| Physical activity | |  | 0.014 | | | -0.103 | | 0.131 | | 0.809 |
| Sleep duration | |  | -0.014 | | | -0.117 | | 0.088 | | 0.781 |
| Self-rated health | |  | -0.016 | | | -0.095 | | 0.064 | | 0.702 |
| Change score self-rated health | |  | -0.059 | | | -0.130 | | 0.011 | | 0.098 |
| Body fat percentage | |  | -0.043 | | | -0.172 | | 0.085 | | 0.508 |
| CRP*body fat percentage | |  | 0.042 | | | 0.008 | | 0.075 | | 0.014* |
| CRP Model B | | 262 |  | | |  | |  | |  |
| CRP | |  | 0.049 | | | 0.026 | | 0.072 | | <0.001* |
| Baseline HSCL-10 | |  | 0.615 | | | 0.472 | | 0.757 | | <0.001* |
| Physical activity | |  | 0.066 | | | -0.056 | | 0.189 | | 0.286 |
| Sleep duration | |  | -0.019 | | | -0.120 | | 0.083 | | 0.720 |
| Self-rated health | |  | -0.023 | | | -0.100 | | 0.053 | | 0.550 |
| Change score self-rated health | |  | -0.065 | | | -0.134 | | 0.005 | | 0.069 |
| CRP*physical activity | |  | -0.045 | | | -0.076 | | -0.013 | | 0.006* |
| TGF-α Model 2 | | 279 |  | | |  | |  | |  |
| TGF-α | |  | 0.123 | | | 0.042 | | 0.205 | | 0.004* |
| Baseline HSCL-10 | |  | 0.621 | | | 0.502 | | 0.739 | | <0.001* |
| TWEAK Model | | 274 |  | | |  | |  | |  |
| TWEAK | |  | 0.210 | | | -0.011 | | 0.432 | | 0.062 |
| Baseline HSCL-10 | |  | 0.598 | | | 0.464 | | 0.733 | | <0.001* |
| Physical activity | |  | -0.030 | | | -0.143 | | 0.083 | | 0.600 |
| Sleep duration | |  | 3.679 | | | 0.574 | | 6.785 | | 0.020* |
| Self-rated health | |  | 0.043 | | | -0.115 | | 0.030 | | 0.251 |
| Change score self-rated health | |  | 0.070 | | | -0.140 | | -0.001 | | 0.047* |
| TWEAK*Sleep duration | |  | -0.046 | | | -0.750 | | -0.062 | | 0.021* |

*B*: Unstandardized beta

*Statistically significant with a p-value cutoff of 0.05

CRP: C-reactive protein

TGF-α: Transforming growth factor alpha

TWEAK: Tumor necrosis factor-like weak inducer of apoptosis (O43508: TNF-like weak inducer of apoptosis within limits of detection)
