# Supplementary material for: C-Reactive Protein and TGF-α Predict Psychological Distress at Two Years of Follow-Up in Healthy Adolescent Boys: The Fit Futures Study
Source: Front Psychol. 2022 Mar 11;13:823420. doi: 10.3389/fpsyg.2022.823420 (PMC8963454; doi:10.3389/fpsyg.2022.823420)
Supplement: Supplementary file 3 [file Table_3.DOCX]

**Supplementary table 3:** *Crude and adjusted* *associations between baseline inflammatory proteins and the six depressive symptoms from HSCL-10 at follow-up, assessed by linear regressions. The results are presented for girls and boys, respectively.* *Fit futures 2010-2011 and 2012-2013.*

| Girls |  | |  | |  | | | |
| --- | --- | --- | --- | --- | --- | --- | --- | --- |
|  | | | | 95 % CI | | |  |  |
|  | *n* | *B* | | Lower | | Upper | *p*-value | R^2^ change |
| CRP |  |  | |  | |  |  |  |
| Model 1 | 330 | 0.017 | | -0.008 | | 0.043 | 0.174 | 0.006 |
| Model 2 | 330 | 0.016 | | -0.005 | | 0.036 | 0.127 | **0.342** |
| Model 3 | 324 | 0.004 | | -0.017 | | 0.024 | 0.722 | **0.070** |
| IL-6 |  |  | |  | |  |  |  |
| Model 1 | 331 | 0.086 | | -0.039 | | 0.211 | 0.179 | 0.006 |
| Model 2 | 331 | 0.048 | | -0.054 | | 0.151 | 0.353 | **0.336** |
| Model 3 | 325 | 0.009 | | -0.094 | | 0.112 | 0.866 | **0.074** |
| TGF-α |  |  | |  | |  |  |  |
| Model 1 | 331 | 0.083 | | -0.052 | | 0.217 | 0.227 | 0.005 |
| Model 2 | 331 | 0.104 | | -0.005 | | 0.213 | 0.060 | **0.343** |
| Model 3 | 325 | 0.076 | | -0.029 | | 0.181 | 0.156 | **0.072** |
| TRANCE (TNF) |  |  | |  | |  |  |  |
| Model 1 | 331 | 0.007 | | -0.125 | | 0.138 | 0.922 | <0.001 |
| Model 2 | 331 | 0.048 | | -0.059 | | 0.155 | 0.374 | **0.341** |
| Model 3 | 325 | 0.027 | | -0.081 | | 0.136 | 0.620 | **0.075** |
| TWEAK (TNF) |  |  | |  | |  |  |  |
| Model 1 | 331 | 0.118 | | -0.128 | | 0.363 | 0.345 | 0.003 |
| Model 2 | 331 | 0.149 | | -0.050 | | 0.349 | 0.141 | **0.342** |
| Model 3 | 325 | 0.184 | | -0.015 | | 0.384 | 0.070 | **0.078** |
| Boys |  | |  | |  | | | |
|  | | | | 95 % CI | | |  |  |
|  | *n* | *B* | | Lower | | Upper | *p*-value | R^2^ change |
| CRP |  |  | |  | |  |  |  |
| Model 1 | 266 | 0.025 | | 0.002 | | 0.047 | 0.030* | **0.018** |
| Model 2 | 266 | 0.031 | | 0.012 | | 0.050 | 0.002* | **0.249** |
| Model 3 | 262 | 0.029 | | 0.010 | | 0.049 | 0.004* | 0.011 |
| IL-6 |  |  | |  | |  |  |  |
| Model 1 | 279 | 0.060 | | -0.049 | | 0.169 | 0.278 | 0.004 |
| Model 2 | 279 | 0.074 | | -0.019 | | 0.168 | 0.118 | **0.263** |
| Model 3 | 274 | 0.077 | | -0.016 | | 0.170 | 0.105 | 0.015 |
| TGF-α |  |  | |  | |  |  |  |
| Model 1 | 279 | 0.154 | | 0.036 | | 0.272 | 0.010* | **0.024** |
| Model 2 | 279 | 0.127 | | 0.026 | | 0.229 | 0.014* | **0.253** |
| Model 3 | 274 | 0.125 | | 0.023 | | 0.226 | 0.017* | 0.014 |
| TRANCE (TNF) |  |  | |  | |  |  |  |
| Model 1 | 279 | -0.017 | | -0.148 | | 0.115 | 0.802 | <0.001 |
| Model 2 | 279 | 0.002 | | -0.112 | | 0.115 | 0.979 | **0.261** |
| Model 3 | 274 | 0.008 | | -0.106 | | 0.122 | 0.890 | 0.015 |
| TWEAK (TNF) |  |  | |  | |  |  |  |
| Model 1 | 279 | 0.049 | | -0.196 | | 0.295 | 0.693 | 0.001 |
| Model 2 | 279 | 0.102 | | -0.110 | | 0.313 | 0.345 | **0.263** |
| Model 3 | 274 | 0.104 | | -0.107 | | 0.316 | 0.334 | 0.015 |

B: Unstandardized beta

*Statistically significant with a p-value of 0.05

Bold: significant R2 change

CRP: C-reactive protein

IL6-α: Interleukin 6 alpha

TGF-α: Transforming growth factor alpha

TRANCE: Tumor Necrosis Factor-related activation-induced cytokine (O14788: TNF-related activation-induced cytokine within limits of detection)

TWEAK: Tumor necrosis factor-like weak inducer of apoptosis (O43508: TNF-like weak inducer of apoptosis within limits of detection)

Model 1: Crude analysis with the respective inflammatory marker

Model 2: Model 1 + baseline 6 depressive items from HSCL-10

Model 3 girls: Model 2 + current infection, medication intake, sleep duration, hormonal contraceptives, smoking, self-rated health, and change score self-rated health (inclusion criteria 0.1 from simple regressions)

Model 3 boys: Model 2 + physical activity, sleep duration, self-rated health, and change score self-rated health (inclusion criteria 0.1 from simple regressions)
